# Comparative Preclinical Evaluation of Peptide-Based Chelators for the Labeling of DARPin G3 with ^99m^Tc for Radionuclide Imaging of HER2 Expression in Cancer

**DOI:** 10.3390/ijms232113443

**Published:** 2022-11-03

**Authors:** Mariia Larkina, Evgenii Plotnikov, Ekaterina Bezverkhniaia, Yulia Shabanova, Maria Tretyakova, Feruza Yuldasheva, Roman Zelchan, Alexey Schulga, Elena Konovalova, Anzhelika Vorobyeva, Javad Garousi, Torbjörn Gräslund, Mikhail Belousov, Vladimir Tolmachev, Sergey Deyev

**Affiliations:** 1 Research Centrum for Oncotheranostics, Research School of Chemistry and Applied Biomedical Sciences, Tomsk Polytechnic University, 634050 Tomsk, Russia; 2 Department of Pharmaceutical Analysis, Siberian State Medical University, 634050 Tomsk, Russia; 3 Department of Nuclear Medicine, Cancer Research Institute, Tomsk National Research Medical Center, Russian Academy of Sciences, 634009 Tomsk, Russia; 4 Molecular Immunology Laboratory, Shemyakin & Ovchinnikov Institute of Bioorganic Chemistry, Russian Academy of Sciences, 117997 Moscow, Russia; 5 Department of Immunology, Genetics and Pathology, Uppsala University, 75185 Uppsala, Sweden; 6 Department of Protein Science, School of Engineering Sciences in Chemistry, Biotechnology and Health, KTH Royal Institute of Technology, 11417 Stockholm, Sweden

**Keywords:** radionuclide, HER2, DARPin, SPECT, ^99m^Tc, imaging, cancer

## Abstract

Non-invasive radionuclide imaging of human epidermal growth factor receptor type 2 (HER2) expression in breast, gastroesophageal, and ovarian cancers may stratify patients for treatment using HER2-targeted therapeutics. Designed ankyrin repeat proteins (DARPins) are a promising type of targeting probe for radionuclide imaging. In clinical studies, the DARPin [^99m^Tc]Tc-(HE)_3_-G3 labeled using a peptide-based chelator His-Glu-His-Glu-His-Glu ((HE)_3_), provided clear imaging of HER2 expressing breast cancer 2–4 h after injection. The goal of this study was to evaluate if the use of cysteine-containing peptide-based chelators Glu-Glu-Glu-Cys (E_3_C), Gly-Gly-Gly-Cys (G_3_C), and Gly-Gly-Gly-Ser-Cys connected via a (Gly-Gly-Gly-Ser)_3_-linker (designated as G3-(G_3_S)_3_C) would further improve the contrast of imaging using ^99m^Tc-labeled derivatives of G3. The labeling of the new variants of G3 provided a radiochemical yield of over 95%. Labeled G3 variants bound specifically to human HER2-expressing cancer cell lines with affinities in the range of 1.9–5 nM. Biodistribution of [^99m^Tc]Tc-G3-G_3_C, [^99m^Tc]Tc-G3-(G_3_S)_3_C, and [^99m^Tc]Tc-G3-E_3_C in mice was compared with the biodistribution of [^99m^Tc]Tc-(HE)_3_-G3. It was found that the novel variants provide specific accumulation in HER2-expressing human xenografts and enable discrimination between tumors with high and low HER2 expression. However, [^99m^Tc]Tc-(HE)_3_-G3 provided better contrast between tumors and the most frequent metastatic sites of HER2-expressing cancers and is therefore more suitable for clinical applications.

## 1. Introduction

Oncogenesis and progression of many carcinomas are tightly associated with overexpression of some tyrosine kinase receptors. A spectacular example of such kinases is human epidermal growth factor receptor type 2 (HER2), which plays a critical role as a driving oncoprotein in many malignancies [1]. This receptor is overexpressed in many cases of breast, esophagus, gastroesophageal, ovarian, and other cancers. Therapeutics which are dependent on the specific recognition of HER2 include monoclonal antibodies, antibody-drug conjugates, and tyrosine kinase inhibitors. These therapeutics are routinely used for the therapy of breast [2] and gastroesophageal [3] carcinomas. HER2-targeted therapeutics are also under clinical evaluation for the treatment of ovarian [4], non-small cell lung [5], uterine [6], and urothelial [7] cancers. Accurate determination of the level of HER2 expression in tumors is a precondition for routine use and further development of such therapies. In the case of insufficiently high target expression, an antitumor effect could not be expected, but patients would be exposed to the risk of side effects, especially when targeted drugs and toxins are used. The major challenge with the use of HER2-targeting therapeutics is the heterogeneity of HER2 expression in malignant tumors. The routine method for determination of HER2 expression is based on biopsies [8,9]. However, biopsy sampling is complicated when multiple metastases are present due to the invasiveness of this procedure. The biopsy-based methodology is also suboptimal for detection of alteration of the HER2 expression level, which can happen in over 20% of all cases after neoadjuvant therapy [10]. To overcome the problem with the invasiveness of biopsies, in vivo radionuclide molecular imaging of HER2 expression has been proposed [11,12,13]. A comparison of preclinical [14] and some clinical [13] data concerning different types of probes for molecular visualization of HER2 (antibodies, antibody fragments, scaffold proteins, rationally designed peptides, and aptamers) suggests that the most promising types of probes are the engineered scaffold proteins. Compared to other targeting agents, scaffold proteins offer a shorter time between injections and imaging of HER2. Furthermore, they provide higher imaging contrast than, e.g., labeled antibodies, which creates the potential for a higher sensitivity of imaging diagnostics.

Designed Ankyrin Repeat Proteins are engineered affinity proteins based on a scaffold developed by Prof. Plückthun et al [15]. A typical DARPin monomer has a molecular weight of either 14 or 18 kDa. The small size of DARPins is an advantage in the development of imaging probes because it facilitates their localization in tumors. Another important feature of DARPins is their stability in a broad range of pH and temperatures, allowing for a wide range of labeling techniques. Selection of DARPins, which bind with strong affinity and high specificity to different proteins, is possible using ribosome display, phage display, and yeast display techniques [15]. DARPins with high affinity for HER2 were selected using ribosome display and demonstrated an apparent potential for tumor targeting [16,17,18]. The feasibility of radionuclide imaging of HER2 expression in human tumor xenografts in mice using DARPins labeled with ^111^In and ^125^I has been demonstrated by Goldstein et al. [19]. Further investigations demonstrated that the DARPin G3 is the best variant for the development of agents for radionuclide imaging [20].

Radionuclide molecular imaging can be performed using two different methods, single photon computed tomography (SPECT) or positron emission tomography (PET). Both methods provide 3D-reconstruction of activity in vivo but differ in the basic physics of image acquisition. This requires the use of different nuclides. Generally, PET provides better resolution, sensitivity, and accuracy in measurement of activity than standard PET, but is appreciably more expensive. Accordingly, PET facilities are relatively abundant and available in Western Europe and Northern America. Many countries in Asia, Africa, and Southern America have access mainly to SPECT. Thus, development of imaging agents for SPECT might facilitate implementation of personalized treatment of HER2-positive cancer in a larger part of the world. These considerations prompted us to develop DARPin-based probes for SPECT imaging.

The most frequently used radionuclide for SPECT imaging is ^99m^Tc [21]. This radionuclide has a favorable emission profile, providing good spatial resolution and low absorbed dose to patients. A half-life of 6 h permits imaging acquisition up to 24 h post-injection. ^99m^Tc is produced from generators containing ^99^Mo (half-life 65.9 h), which might be delivered to distant hospitals and provide ^99m^Tc up to 2 weeks. Thus, ^99m^Tc is an attractive label for SPECT-based radionuclide imaging. An interesting feature of ^99m^Tc is that it can be conjugated to proteins and peptides using peptide-based chelators, where amide nitrogens and/or functional groups from side chains of certain amino acids act as donor atoms. Such chelators might be incorporated into targeting proteins by genetic engineering. The targeting proteins might be produced in a single biotechnological process, without the need to conjugate a chelator and perform additional purification steps. This would simplify and make the whole production process cheaper.

**Figure 1:**
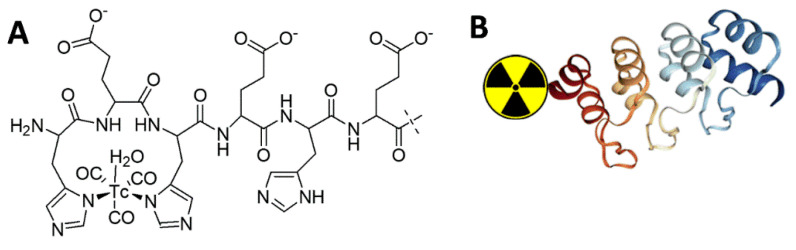
Schematic drawings of (**A**) complex of [^99m^Tc]Tc(CO)_3_^+^ with (HE)_3_-tag and (**B**) its position at N-terminus of the DARPin G3.

The initial development of ^99m^Tc-labeled DARPin-based imaging probes was based on the use of a technetium tricarbonyl ([^99m^Tc]Tc(CO)_3_^+^) core in combination with a hexahistidine-containing peptide-based chelator (histidine tags or His_6_-tags) [20,22]. An attractive feature of this approach is that the histidine tags can be used not only for stable labeling of proteins with technetium but also for their purification using immobilized metal-ion affinity chromatography after recombinant production [23]. While these tracers demonstrated good visualization of HER2-expressing xenografts, there was a high accumulation in the liver. During our work with other scaffold proteins, affibody molecules, we found that a substitution of every second histidine in the His_6_-tag by glutamate resulted in a (HE)_3_-tag. It also allows for stable labeling using a ^99m^Tc(CO)_3_ core, but provides appreciably lower hepatic uptake [24]. Evaluation of this tag demonstrated that positioning at the N-terminus of the HER2-targeting DARPin G3 resulted in three-fold reduction of hepatic uptake, compared to the uptake of other His_6_-tag-containing variants [25]. This variant, designated as [^99m^Tc]Tc-(HE)_3_-G3 (Figure 1), was evaluated in a Phase I clinical trial [26]. The clinical data demonstrated that the injections of [^99m^Tc]Tc-(HE)_3_-G3 were safe and provided low dose burden on the patients. Imaging using [^99m^Tc]Tc-(HE)_3_-G3 permitted discrimination between HER2-positive and HER2-negative breast cancer tumors.

**Figure 2:**
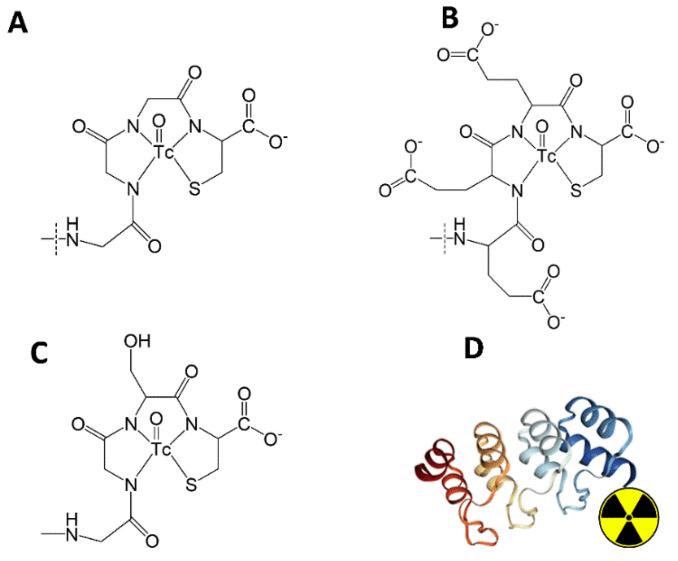
Schematic drawings of oxotechnetium complexes with chelating moieties of (**A**) G3-G_3_C, (**B**) G3-E_3_C, (**C**) G3-(G_3_S)_3_C, and (**D**) their position at the C-terminus of the DARPin G3.

A possible disadvantage of [^99m^Tc]Tc-(HE)_3_-G3 for clinical translation is the use of a two-step labeling procedure, which includes conversion of [^99m^Tc]Tc-pertechnetate into the [^99m^Tc]Tc(CO)_3_^+^-core and then conjugation of [^99m^Tc]Tc(CO)_3_^+^ to the targeting DARPin. Another issue is the requirement for size-exclusion chromatography to purify the labeled [^99m^Tc]Tc-(HE)_3_-G3 from the reaction mixture components. Apparently, development of a single-step labeling procedure, which would not require purification, would make the labeling more robust and streamline clinical implementation [21]. Experience in the labeling of affibody molecules and ADAPTs suggests that such a protocol might be achieved by the use of a cysteine-containing peptide-based chelator placed on the C-terminus of a scaffold protein [27,28,29]. In this case, an SN_3_ chelator is formed by the thiol group of cysteine and amide nitrogens of amino acids placed at the N-terminus (Figure 2). Importantly, the selection of amino acids in such chelators influences the intracellular retention of ^99m^Tc after internalization of the targeting protein by cells in tumors and in normal tissue. The use of charged amino acids, e.g., glutamate or lysine, results in so-called residualizing labels, i.e., providing a strong intracellular retention of activity [27]. They enable an improved intracellular retention of activity by cancer cells but also increase the retention by the kidneys in the case of renal re-absorption. In addition, the incorporation of glutamates reduced the hepatic uptake of affibody molecules, but this effect should be weighed against the increased retention of intracellular activity in the liver. Glycine- and serine-containing variants are non-residualizing, i.e., they diffuse from cells after proteolytic degradation of the targeting proteins in the lysosomal compartments. This allows for a reduction in the renal and hepatic retention of ^99m^Tc. Thus, the selection of an optimal cysteine-containing peptide-based chelator may provide the highest possible uptake of activity in tumors and the lowest possible uptake in normal tissues, i.e., improve the contrast of imaging. The problem is that in vivo interactions of any targeting protein are difficult to predict. Therefore, in vivo studies concerning the influence of chelators on biodistribution are necessary to select the best chelator.

To evaluate the impact of different chelators on the biodistribution of ^99m^Tc-labeled DARPins, we created a small library, which included a variant containing a Glu-Glu-Glu-Cys chelator providing a residualizing label (designated as G3-E_3_C), a variant containing Gly-Gly-Gly-Cys chelator providing a non-residualizing label (designated as G3-G_3_C), and a variant with a Gly-Gly-Gly-Ser-Cys chelator connected with the C-terminus of G3 via a -(Gly-Gly-Gly-Ser)-linker (designated as G3-(G_3_S)_3_C) (Figure 2).

The goal of the study was to compare the affinity, cellular processing, biodistribution, and in vivo targeting properties of these variants with each other and with the best previous variant, [^99m^Tc]Tc-(HE)_3_-G3.

## 2. Results

### 2.1. Protein Production and Characterization

**Figure 3:**
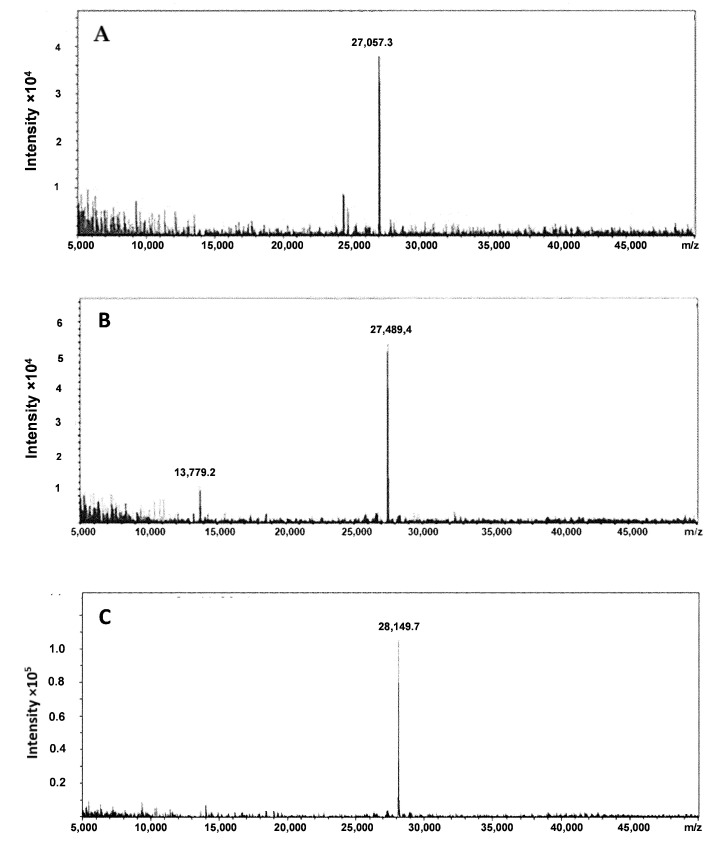
Deconvolution of mass spectra of (**A**) G3-G_3_C, (**B**) G3-E_3_C, and (**C**) G3-(G_3_S)_3_C.

Three new DARPin G3 variants having amino acid-containing sequences G_3_C, E_3_C, and (G_3_S)_3_C at the C-terminus were efficiently produced using *E. coli* and were subsequently purified. The authenticity and purity of the DARPin G3 variants were confirmed using LC-MS. The purity of the proteins was close to 100%. The results of the mass spectrometry analysis are presented in Figure 3 and Table 1. Deconvolution of the mass spectrometry data showed that the proteins were primarily present in a dimeric form (Figure 3). The molecular weights of the corresponding monomers are in agreement with the calculated values with an accuracy better than 0.5 Da (Table 1).

### 2.2. Radiolabeling

Initially, the ^99m^Tc-labeling of the new variants (G3-G_3_C, G3-E_3_C and G3-(G_3_S)_3_C) was performed without pre-reduction of spontaneously formed intermolecular disulfide bonds between cysteines. The radiochemical yield was 30–80% and, after purification using a NAP-5 column, the radiochemical purity was 80–90%. When the DARPins were reduced before labeling with dithiothreitol, the radiochemical yield was 97–99%. The radiochemical purity was close to 100% after purification (Table 2). For the variants G3-G_3_C and G3-(G_3_S)_3_C, an incubation time of no more than 30 min was required to obtain the maximum yield. A somewhat longer time, 60 min, was required to obtain the maximum radionuclide incorporation into G3-E_3_C. The radiochemical yield after 30 min was only about 80%.

The results of the in vitro stability test demonstrated high stability of all variants during storage in PBS. The radiochemical purity was ≥97% after 6 h incubation. However, the radiochemical purity was reduced to 92–95% after 2–4 h incubation in PBS on several occasions. This indicated that a re-oxidation of ^99m^Tc might play a role in its release. To avoid this, we added 20 µg/mL tin (II) chloride as an antioxidant to the purification buffer. The release of ^99m^Tc did not occur in this case (radiochemical purity ≥ 98%, 6 h). Tin (II) chloride was henceforth included in the formulation of ^99m^Tc-labeled G3-variants for in vivo studies.

### 2.3. In Vitro Studies

**Figure 4:**
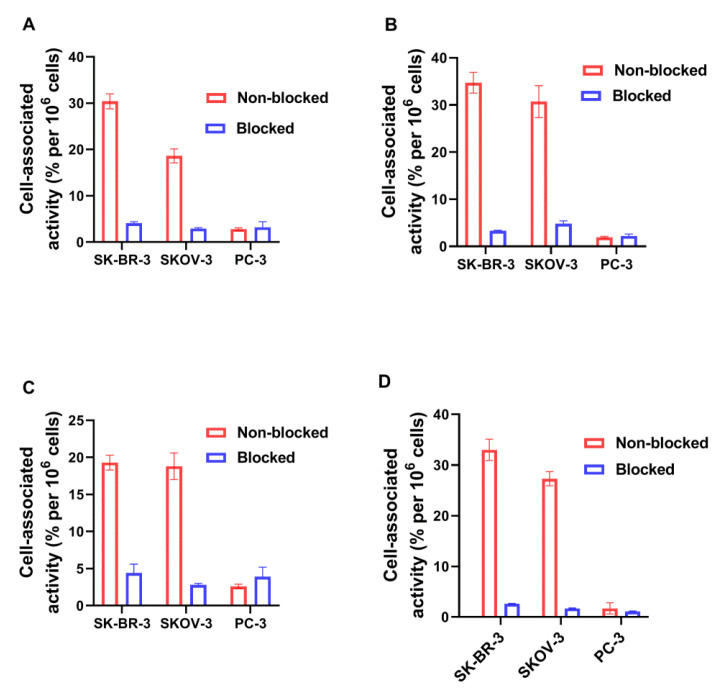
Binding specificity of radiolabeled G3 variants to HER2-expressing SK-BR-3, SKOV-3, and PC-3 cells in vitro. (**A**) [^99m^Tc]Tc-G3-G_3_C, (**B**) [^99m^Tc]Tc-G3-(G_3_S)_3_C, (**C**) [^99m^Tc]Tc-G3-E_3_C, and (**D**) [^99m^Tc]Tc-(HE)_3_-G3. For blocking, a 100-fold molar excess of non-labeled G3 was added to the blocked groups. The final concentration of radiolabeled compounds was 1 nM. The data are presented as the mean from three samples ±SD.

The blocking of the receptors by adding a large excess of non-labeled DARPin-G3 significantly decreased the binding of the radiolabeled DARPin-G3 variants to cell lines with high HER2 expression (SKOV-3 and SK-BR-3, *p* ˂ 0.001) (Figure 4). The level of binding to SKOV-3 and SK-BR-3 was significantly (*p* ˂ 0.05) higher than the level of binding to PC-3 cells. These data suggest that the binding of all variants of labeled DARPin G3 to HER2-expressing cells was receptor mediated.

**Figure 5:**
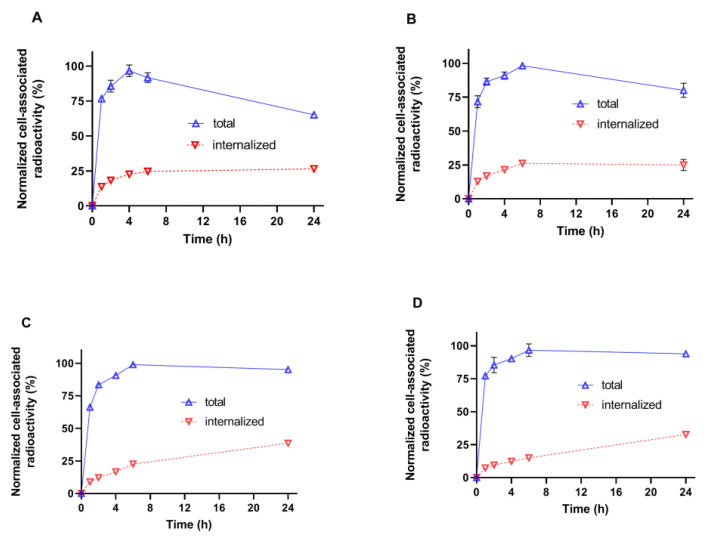
Cellular processing of radiolabeled G3 variants by HER2-expressing SKOV-3 cells in vitro. (**A**) [^99m^Tc]Tc-G3-G_3_C, (**B**) [^99m^Tc]Tc-G3-(G_3_S)_3_C, (**C**) [^99m^Tc]Tc-G3-E_3_C, and (**D**) [^99m^Tc]Tc-(HE)_3_-G3. The data are presented as the average of three samples ±SD. Some error bars are not seen because they are smaller than point symbols.

The validation of the internalization test for the new variants of ^99m^Tc-labeled DARPins showed that less than 5% of the activity was associated with cells after 2 h incubation on ice and subsequent acid wash. The results of the internalization assays are presented in Figure 5. The common feature of all tested variants was the relatively low amount of internalized activity. However, the pattern was somewhat different. In the case of the [^99m^Tc]Tc-G3-G_3_C and [^99m^Tc]Tc-G3-(G_3_S)_3_C, the amount of internalized activity reached a plateau by 6 h after the start of incubation. At the same time, the total cell-associated activity reached a maximum by 4–6 h and declined thereafter. In the case of glutamate-containing chelators, i.e., [^99m^Tc]Tc-G3-E_3_C and [^99m^Tc]Tc-(HE)_3_-G3, the internalized activity was continuously increasing and there was no pronounced decrease of the cell-associated activity during the incubation.

**Figure 6:**
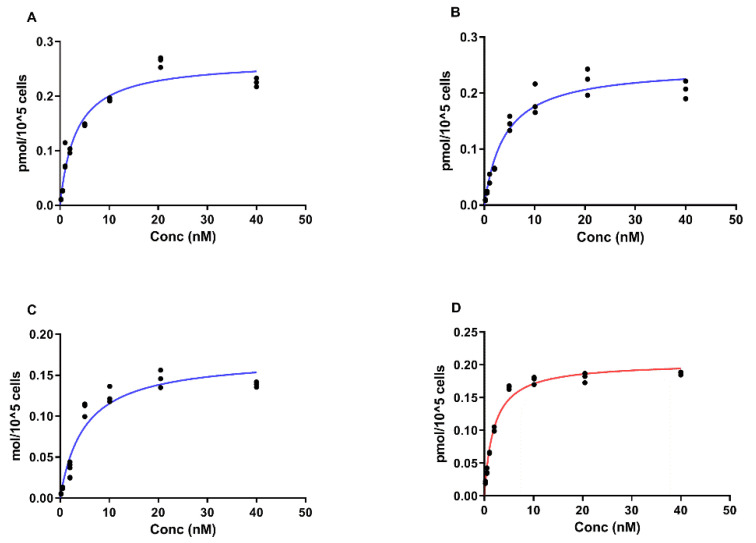
Determination of equilibrium dissociation constants (K_D_) of radiolabeled G3 variants by HER2-expressing SKOV-3 cells in vitro. (**A**) [^99m^Tc]Tc-G3-G_3_C, (**B**) [^99m^Tc]Tc-G3-(G_3_S)_3_C, (**C**) [^99m^Tc]Tc-G3-E_3_C, and (**D**) [^99m^Tc]Tc-(HE)_3_-G3. The data are presented as measurements from three samples for each concentration. For some concentrations the points are overlapping.

The results of a saturation assay are presented in Figure 6 and Table 3. A saturable character of binding of [^99m^Tc]Tc-labeled G3 variants to HER2-overexpressing SKOV-3 cells was clearly observed (Figure 6). Fitting the saturation binding curves for four [^99m^Tc]Tc-labeled G3 variants showed that the K_D_ for binding to HER2 on SKOV-3 cells had a value in the range between 1.9 and 5.0 nM. The [^99m^Tc]Tc-(HE)_3_-G3 variant had significantly (*p* ˂ 0.05) stronger affinity (lower K_D_) than the other variants (Table 3). The number of binding sites (B_max_) was determined to be 1.03–1.59 × 10^6^ receptor sites per cell.

### 2.4. In Vivo Studies

**Figure 7:**
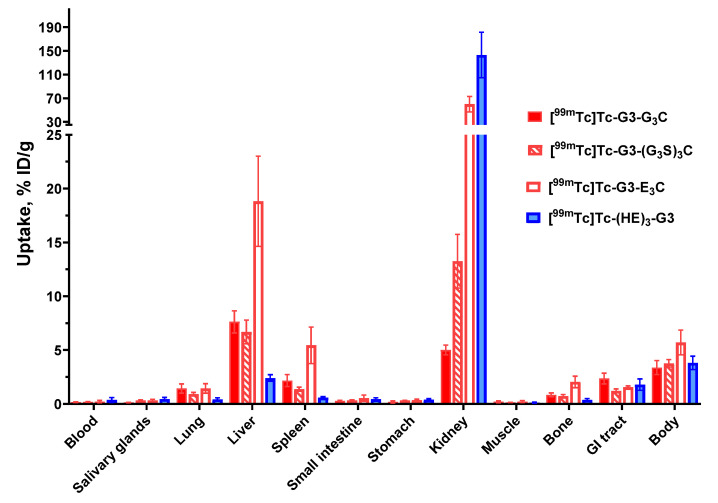
Comparative biodistribution of ^99m^Tc-labeled G3 variants 4 h post-injection (p.i.) in CD1 mice. Data are presented as mean %ID/g ± SD for four mice. Data for the rest of the GI tract with contents and the rest of the body are presented as %ID per whole sample.

Initially, the biodistribution of the G3 variants, labeled with ^99m^Tc, was evaluated in a single batch of CD1 mice at 4 h after injection (Figure 7). A common feature of all variants was a low retention of activity in the blood, muscles, and bones. Apparently, excretion via bile played a minor role, as the activity in the gastrointestinal tract with content was low. Low levels of activity accumulation in the stomach and salivary glands suggest that there was no release of [^99m^Tc]Tc-pertechnetate, i.e., that all labels were stable in vivo. The use of different peptide-based chelators had a very strong influence on accumulation in the kidneys, liver, and spleen. The [^99m^Tc]Tc-(HE)_3_-G3 variant had the highest retention of activity in the kidneys. The uptake of [^99m^Tc]Tc-G3-E_3_C in the kidneys was 2.4-fold lower than the uptake of [^99m^Tc]Tc-(HE)_3_-G3, but still much higher than the uptake of [^99m^Tc]Tc-G3-G_3_C and [^99m^Tc]Tc-G3-(G_3_S)_3_C. The GGGC-containing variant provided significantly (*p* < 0.05) lower uptake in the kidneys than any of the other variants. Hepatic uptake of [^99m^Tc]Tc-G3-E_3_C (18.8 ± 4.2 %ID/g) was the highest, and the uptake of [^99m^Tc]Tc-(HE)_3_-G3 (2.4 ± 0.3 %ID/g) was the lowest. Hepatic uptakes of [^99m^Tc]Tc-G3-G_3_C and [^99m^Tc]Tc-G3-(G_3_S)_3_C had intermediate values, and there was no significant difference between liver uptakes of these constructs. Additionally, the [^99m^Tc]Tc-(HE)_3_-G3 variant had the lowest uptake in the lungs, spleen, and bone (*p* < 0.05). Taking into account the unfavorably high hepatic uptake, the [^99m^Tc]Tc-G3-E_3_C variant was excluded from further in vivo evaluations.

**Figure 8:**
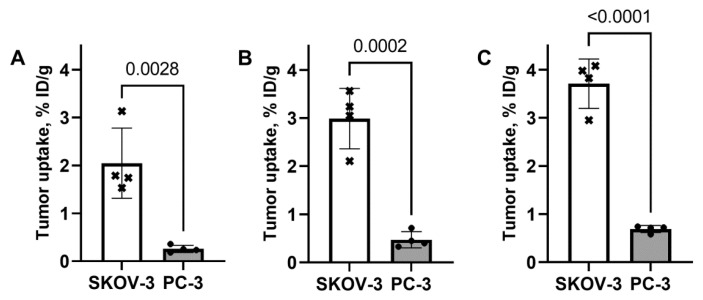
Uptake of [^99m^Tc]Tc-G3-G_3_C (**A**), [^99m^Tc]Tc-G3-(G_3_S)_3_C (**B**), and [^99m^Tc]Tc-(HE)_3_-G3 (**C**) in SKOV-3 (high HER2 expression) and PC-3 (low HER2 expression) xenografts in Nu/j mice 4 h p.i. Data are presented as mean %ID/g ± SD for four mice.

A comparison of [^99m^Tc]Tc-G3-G_3_C, [^99m^Tc]Tc-G3-(G_3_S)_3_C, and [^99m^Tc]Tc-(HE)_3_-G3 uptake in SKOV-3 xenografts with high HER2 expression and PC-3 with low HER2 expression is presented in Figure 8. The uptake in SKOV-3 xenografts was significantly (*p* < 0.005, one-way ANOVA) higher than in PC-3 xenografts (Figure 8). This shows that the level of the uptake correlates with the HER2 expression level for all variants. The uptake of the [^99m^Tc]Tc-G3-G_3_C variant in SKOV-3 xenografts was significantly (*p* < 0.05, one-way ANOVA) lower than the uptake of the [^99m^Tc]Tc-(HE)_3_-G3 variant. The uptakes of [^99m^Tc]Tc-G3-G_3_C and [^99m^Tc]Tc-G3-(G_3_S)_3_C did not differ significantly (*p* > 0.05, one-way ANOVA) in SKOV-3 xenografts at 4 h after injection.

**Figure 9:**
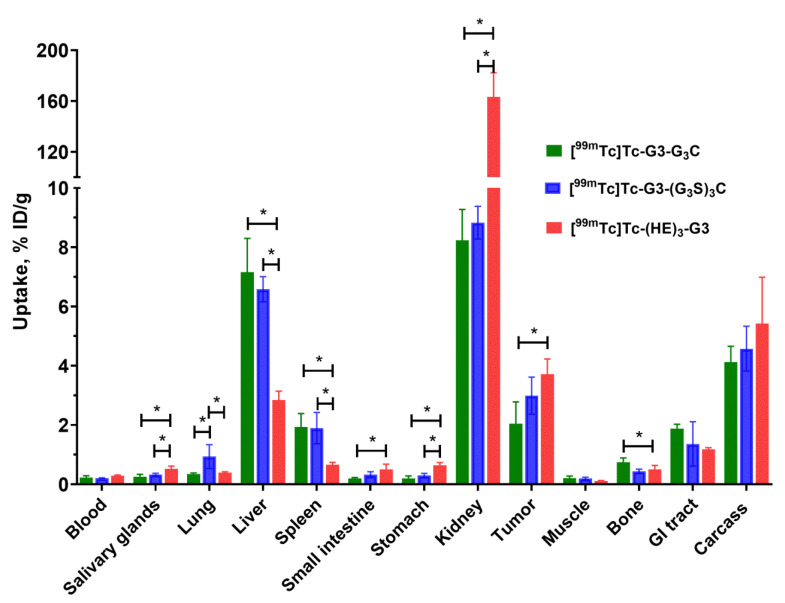
Comparative biodistribution of [^99m^Tc]Tc-G3-G_3_C, [^99m^Tc]Tc-G3-(G_3_S)_3_C, and [^99m^Tc]Tc-(HE)_3_-G3 at 4 h post-injection (p.i.) in Nu/j mice bearing HER2-expressing SKOV-3 xenografts. Data are presented as mean %ID/g ± SD for four mice. Data for the rest of the GI tract with contents and the rest of the body are presented as %ID per whole sample. Asterisks mark a significant difference (*p* < 0.05, one-way ANOVA).

A side-by-side comparison of the biodistribution of [^99m^Tc]Tc-G3-G_3_C, [^99m^Tc]Tc-G3-(G_3_S)_3_C, and [^99m^Tc]Tc-(HE)_3_-G3 in Nu/j mice bearing SKOV-3 xenografts is shown in Figure 9. The pattern of biodistribution of the labeled proteins was similar to their pattern of biodistribution in CD1 mice. There was a significant difference in the uptake of these variants in several normal tissues. All variants demonstrated predominantly renal clearance. As expected, [^99m^Tc]Tc-(HE)_3_-G3 demonstrated high retention of activity in kidneys (163 ± 19 %ID/g in Nu/j mice bearing SKOV-3 xenografts). The uptake of [^99m^Tc]Tc-(HE)_3_-G3 in liver and spleen was significantly (*p* < 0.05, one-way ANOVA) lower than the uptake of constructs with cysteine-containing chelators. The hepatic uptakes of [^99m^Tc]Tc-G3-G_3_C and [^99m^Tc]Tc-G3-(G_3_S)_3_C had intermediate values, and there was no significant difference between liver uptakes of these constructs. Additionally, the [^99m^Tc]Tc-(HE)_3_-G3 variant had the lowest uptake in lungs, spleen, and bone (*p* < 0.05, one-way ANOVA).

**Figure 10:**
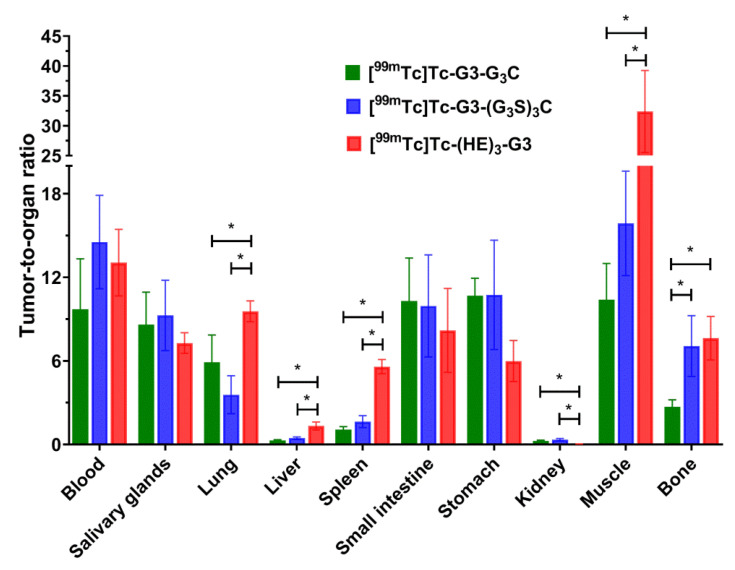
Tumor-to-organ ratios of [^99m^Tc]Tc-G3-G_3_C, [^99m^Tc]Tc-G3-(G_3_S)_3_C, and [^99m^Tc]Tc-(HE)_3_-G3 4 h post-injection (p.i.) in Nu/j mice bearing HER2-expressing SKOV-3 xenografts. Data are presented as mean %ID/g ± SD for four mice. Asterisks mark a significant difference (*p* < 0.05, one-way ANOVA).

The tumor-to-organ ratios for [^99m^Tc]Tc-G3-G_3_C, [^99m^Tc]Tc-G3-(G_3_S)_3_C and [^99m^Tc]Tc-(HE)_3_-G3 at 4 h post-injection in Nu/j mice bearing SKOV-3 xenografts are shown in Figure 10. In direct comparison, [^99m^Tc]Tc-(HE)_3_-G3 provided significantly (*p* < 0.05, one-way ANOVA) higher tumor-to-liver, tumor-to-spleen, tumor-to-lung, and tumor-to-muscle ratios than both variants containing cysteine. Conversely, the tumor-to-kidney ratio was much worse for the (HE)_3_-tag-containing variant.

**Figure 11:**
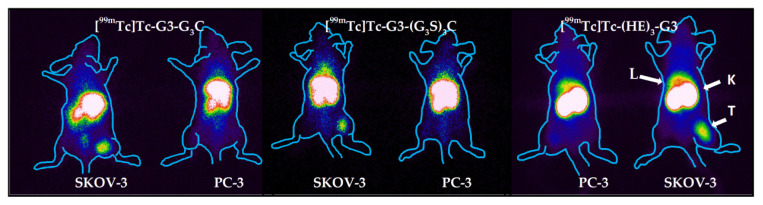
Gamma-camera imaging of HER2 expression in Nu/J mice bearing SKOV-3 and PC-3 xenografts using of [^99m^Tc]Tc-G3-G_3_C, [^99m^Tc]Tc-G3-(G_3_S)_3_C, and [^99m^Tc]Tc-(HE)_3_-G3 4 h pi. (10 μg, 8 MBq). L—liver, K—kidneys, T—tumor. Contours were derived from a digital photograph and superimposed over the images to facilitate interpretation.

The results of the experimental gamma-camera imaging (Figure 11) were in agreement with the biodistribution data. SKOV-3 xenografts were clearly visualized by all ^99m^Tc-labeled DARPins. All radiolabeled G3 variants provided clear discrimination between SKOV-3 xenografts with high HER2 expression and PC-3 xenografts with low HER2 expression. The activity uptake in SKOV-3 xenografts was clearly higher. The renal activity uptake was noticeably higher for [^99m^Tc]Tc-(HE)_3_-G3 compared with the two other variants, but its hepatic uptake was lower. In the case of [^99m^Tc]Tc-G3-G_3_C, there was somewhat elevated activity accumulation in the abdominal area. This is in agreement with the data of the biodistribution measurement, which showed that the activity of [^99m^Tc]Tc-G3-G_3_C in the gastrointestinal tract was higher than the activity of other variants.

## 3. Discussion

Scaffold protein-based probes for radionuclide imaging offer clear advantages in the timing of diagnostics and imaging contrast compared to probes based on full-length monoclonal antibodies. However, the radiolabeled antibodies have been investigated over the last fifty years. The factors influencing imaging using scaffold proteins are much less studied. Moreover, the scaffolds are very different in structure and composition, and knowledge concerning one scaffold might not be directly translated to another. Therefore, careful validation is required to decide if an approach that provides good imaging with one scaffold can be applied to another. This study was dedicated to the evaluation of the use of peptide-based cysteine-containing chelators for labeling of DARPin-based probes with ^99m^Tc.

Our initial attempts to label novel variants were performed without pre-reduction of spontaneously formed intermolecular disulfide bonds between cysteines. We considered that this might be possible because of a strongly reducing environment during labeling. However, these attempts resulted in low radiochemical yield. Moreover, the labeling stability was low, and a label apparently dissociated during purification using size-exclusion chromatography, which resulted in an insufficient radiochemical purity of the labeled probes. This might indicate that the reduced technetium was bound without the involvement of the thiol group of cysteine. Therefore, we performed a pre-reduction of spontaneously formed intermolecular disulfide bonds, which should ensure the availability of thiols for chelate formation. This protocol provided an excellent yield (over 95%) and very good stability (Table 2). The binding of all novel variants to HER2-expressing human cancer cells was saturable and dependent on the HER2 expression level (Figure 4), which indicated that the binding was HER2-specific. The affinity of the new variants was in the single-digit nanomolar range (Figure 6, Table 3), which is sufficient for imaging of molecular targets with high expression, such as HER2 [30]. The pattern of cellular retention of activity during processing of the new DARPin variants after binding to cancer cells differed (Figure 5). A common trait was a low level of internalized activity. However, the cell-bound activity of [^99m^Tc]Tc-G3-G_3_C and [^99m^Tc]Tc-G3-(G_3_S)_3_C reached a maximum after 4–6 h and then declined. Such a pattern is characteristic for non-residualizing labels when labels from internalized and degraded proteins diffuse from cells. The pattern of [^99m^Tc]Tc-G3-E_3_C was similar to the pattern of [^99m^Tc]Tc-(HE)_3_-G3, i.e., slow but continuous increase of internalized activity and a constant level of overall cell-bound activity after 6 h. Such behavior is typical for slowly internalized proteins with residualizing labels. Still, the release of activity of [^99m^Tc]Tc-G3-G_3_C and [^99m^Tc]Tc-G3-(G_3_S)_3_C from cancer cells was slow because of slow internalization, and one could expect a minor effect in activity accumulation in tumors in vivo within 2–4 h after injection, a clinically relevant imaging time. It has to be noted that we performed a validation of the acid wash methods by estimation of internalization after incubation on ice. We found that some cell-associated activity (less than 5%) was still associated with cells after the acid wash. Since internalization is energy-driven, it cannot take place on ice. Most likely, the acid wash failed to remove a small fraction of DARPins, which were strongly bound to HER2 on cellular membranes. This means that the method overestimates the internalized activity, but such overestimation should not exceed 5%.

The biodistribution measurements in normal mice (Figure 7) demonstrated high retention of activity in renal tissue for both [^99m^Tc]Tc-G3-E_3_C and [^99m^Tc]Tc-(HE)_3_-G3. Such biodistribution profiles are consistent with earlier findings for DARPins with residualizing labels in preclinical [20,22,25] and clinical [26] studies. Apparently, DARPins are rapidly internalized after renal reabsorption and the residualizing character of a label ensures its long retention in the kidneys. Application of commonly used methods for blocking renal reabsorption of radiolabeled proteins and peptides (injection of cationic amino acids or Gelofusine) was unsuccessful in the case of labeled DARPins [31]. The renal activity was much lower in the case of [^99m^Tc]Tc-G3-G_3_C and [^99m^Tc]Tc-G3-(G_3_S)_3_C. This is most likely as a result of rapid internalization after reabsorption and release of radiocatabolites of non-residualizing labels. These results are consistent with DARPins labeled with non-residualizing radioiodine having a low renal retention of activity [20,22,32,33]. It is important to note that although high renal reabsorption and retention of an imaging probe is not desirable, a high renal uptake does not prevent detection of bone metastases in the lumbar area using affibody molecules [34]. Indeed the very high liver uptake of [^99m^Tc]Tc-G3-E_3_C was more troublesome. Radionuclide molecular imaging is, first and foremost, meant for target imaging in metastases. Obviously, a tracer has to provide a high contrast between tumors and organs, where metastases are frequently formed. In the case of HER2-expressing cancers, the most frequent metastatic sites are: bone, liver, and lung for breast cancer [35]: liver, peritoneum, lung, and bone for esophageal and gastric cancers [36,37]; and liver, lung, and peritoneum for ovarian cancer [38]. Thus, agents with low uptake in the gastrointestinal tract, liver, bone, and lung are critical for the imaging of HER2 and [^99m^Tc]Tc-G3-E_3_C does not meet this requirement. Generally speaking, the incorporation of negatively charged amino acids reduces hepatic uptake of radiolabeled scaffold proteins. For example, a (HE)_3_-tag reduced the hepatic uptake of ^99m^Tc-labeled HER2 and EpCAM-targeted DARPins in comparison with hexahistidine-containing counterparts when placed on the N-terminus [25,33]. This is in agreement with the data for the DARPin G3 labeled with ^111^In at the C-terminus using a DOTA chelator [19]. Adding a (HE)_3_-tag at the N-terminus of such a construct reduced the hepatic uptake of G3 in comparison with H_6_-tagged and untagged variants. Interestingly, increasing the negative charge of the N-terminus also reduced the hepatic uptake of radiolabeled affibody molecules [39]. It is quite likely that exceeding positive charge locally at the N-terminus of G3 is critical for its hepatic uptake, and it cannot be compensated for by adding negatively charged glutamates at the C-terminus. One possible solution would be to place a cysteine-containing chelator at the C-terminus for labeling and an (HE)_3_-tag at the N-terminus for modification of biodistribution. Such an approach was tested for affibody molecules labeled using oxotechnetium [40]. It appeared that a part of the label was unstably attached to the (HE)_3_-tag, i.e., and the site-specificity of labeling was lost. It is unlikely that such an approach would work for DARPins. The data for high hepatic uptake prompted us to exclude [^99m^Tc]Tc-G3-E_3_C from evaluation in tumor-bearing mice. Most likely, the high hepatic uptake also takes place for [^99m^Tc]Tc-G3-G_3_C and [^99m^Tc]Tc-G3-(G_3_S)_3_C, but in these cases ^99m^Tc is rapidly released from the liver in the same ways as from kidneys. Thus, the biodistribution of G_3_C and (G_3_S)_3_C-containing variants was favorable for HER2-imaging probes. Biodistribution of these variants was compared with the biodistribution of [^99m^Tc]Tc-(HE)_3_-G3 in immunodeficient mice bearing human HER2-expressing tumor xenografts.

In mice with human xenografts, all tested variants demonstrated tumor uptake, which was dependent on the HER2 expression level (Figure 8), which indicates HER2 specific targeting in vivo. Uptake of [^99m^Tc]Tc-(HE)_3_-G3 in HER2-positive SKOV3 xenografts was significantly higher (*p* < 0.05, one-way ANOVA) than the uptake of [^99m^Tc]Tc-G3-G_3_C. This might be explained by a combination of the residualizing behavior of the [^99m^Tc]Tc-(HE)_3_ label ensuring better retention and higher affinity of [^99m^Tc]Tc-(HE)_3_-G3 compared to the affinity of [^99m^Tc]Tc-G3-G_3_C. The uptake in normal organs (Figure 9) was in good agreement with the data for normal mice (Figure 7). As pointed out above, metastases of HER2-expressing cancers are most frequent in the gastrointestinal tract, liver, bone, and lung. To provide high imaging sensitivity, the radioactivity concentration ratios between the tumor and these organs should be as high as possible. [^99m^Tc]Tc-(HE)_3_-G3 provides significantly (*p* < 0.05, one-way ANOVA) higher values of tumor-to-organ ratios for lung and liver than both cysteine-containing variants and higher values for bone than [^99m^Tc]Tc-G3-G_3_C (Figure 9). Thus, [^99m^Tc]Tc-(HE)_3_-G3 is the best variant for clinical translation.

## 4. Materials and Methods

### 4.1. General Materials and Instruments

The molecular weight of the DARPins was measured by liquid chromatography-electrospray ionization-mass spectrometry (LC-ESI-MS) on a 6520 Accurate Q-TOF LC/MS (Agilent). The CRS (Center for Radiopharmaceutical Sciences) kits for the production of tricarbonyl technetium were purchased from the Center for Radiopharmaceutical Sciences (PSI, Villigen, Switzerland; contact e-mail: crs-kit@psi.ch). Instant thin-layer chromatography (iTLC) analysis was performed using iTLC silica gel chromatography paper strips (Aglient Technologies, Inc., Folsom, CA, USA). The radioactivity distribution along iTLC strips was measured using an iTLC-scanner miniGITA Single (Elysia Raytest, Straubenhardt, Germany). Radioactivity was measured using an automated gamma-spectrometer with an NaI(TI) detector Wizard 1480 (Pelkin Elmer, Waltham, MA, USA). For formulation of the injection solution for the in vivo experiments, the radioactivity was measured using a dose calibrator (RIS 1A, Amplituda, Saint Petersburg, Russia) equipped with an ionization chamber. The technetium-99m pertechnetate, [^99m^Tc]TcO_4_, was obtained from a commercial ^99^Mo/^99m^Tc generator, GT-4K (FSUE “Karpov Institute of Physical Chemistry”, Obninsk, Russia). The human cancer cell lines with high expression of HER2, SKOV-3 (ovarian carcinoma) and SK-BR-3 (breast adenocarcinoma), as well as PC-3 (prostate adenocarcinoma) with very low expression of HER2, were purchased from the American Type Culture Collection (ATCC). The cells were cultured in Roswell Park Memorial Institute (RPMI) medium supplemented with 10% fetal bovine serum (FBS), 2 mM L-glutamine, 100 IU/mL penicillin, and 100 µg/mL streptomycin, in a humidified incubator with 5% CO_2_ at 37 °C. The in vitro experiments were performed using 35-mm Petri dishes (Nunclon Delta Surface, ThermoFisher Scientific, Roskilde, Denmark).

Unpaired 2-tailed *t*-tests or ANOVA tests with Bonferroni’s post-hoc analysis were used to determine significant differences (*p <* 0.05). Bonferroni’s post-hoc analysis was selected because the equal variance of the biodistribution data was expected and this method provides a somewhat more stringent threshold for significance than alternative methods of analysis (e.g., Šídák’s analysis). Statistical analysis was carried out using Prism (version 9.0.0 for Windows; GraphPad Software, La Jolla, CA, USA).

### 4.2. Protein Production and Characterization

The DARPin G3 gene nucleotide sequence was deduced from a DARPin G3 amino acid sequence deposited in PDB (accession number PDB: 2JAB) taking into account the codon usage in highly expressed *Escherichia coli* genes with the help of the freely distributed program DNABuilder (http://www.innovationsinmedicine.org/software/DNABuilder/, accessed on 15 January 2019). The gene was assembled by PCR from chemically synthesized oligonucleotides of 50 bp in length, having partially complementary sequences.

The proteins G3-G_3_C, G3-(G_3_S)_3_C and G3-E_3_C were produced in *Escherichia coli* BL21(DE3) (Novagen-EMD Millipore, Madison, WI, USA) as the *C*-terminal extensions of the small ubiquitin related modifier SUMO [41] essentially in the same way as described in [32].

The amino acid sequence for G3-G_3_C was: DLGKKLLEAARAGQDDEVRILMANG-ADVNAKDEYGLPLYLATAHGHLEIVEVLLNGADVNAVDAIGFTPLHLAAFIGHLEIAEVLLKHGADVNAQDKFGKTAFDISIGNGNEDLAEILQKLNGGGGC. The calculated molecular weight is 13,529.3 Da.

The amino acid sequence for G3-E_3_C was: DLGKKLLEAARAGQDDEVRILMANG-ADVNAKDEYGLPLYLATAHGHLEIVEVLLKNGADVNAVDAIGFTPLHLAAFIGHLEIAEVLLKHGADVNAQDKFGKTAFDISIGNGNEDLAEILQKLNGEEEC.

The calculated molecular weight is 13,745.5 Da.

The amino acid sequence for G3-(G_3_S)_3_C was: DLGKKLLEAARAGQDDEVRILMANG-ADVNAKDEYGLPLYLATAHGHLEIVEVLLNGADVNAVDAIGFTPLHLAAFIGHLEIAEVLLKHGADVNAQDKFGKTAFDISIGNGNEDLAEILQKLNGGGSGGGSGGGSC.

The calculated molecular weight is 14,075.8 Da

The amino acid sequence for (HE)_3_-G3 was: MRGSHEHEHEGSDLGKKLLEAARA-GQDDEVRILMANGADVNAKDEYGLTPLYLATAHGHLEIVEVLLKNGADVNAVDAIGFTPLHLAAFIGHLEIAEVLLKHGADVNAQDKFGKTAFDISIGNGNEDLAEILQKLN.

Expression, isolation, and purification of (HE)_3_-G3 was performed according to the methodology described earlier [20]. Protein concentrations were determined by UV spectroscopy using ε_280_ = 2980 M^−1^ cm^−1^ for all proteins. To confirm the purity and authenticity of the novel DARPin G3 variants, they were analyzed by liquid chromatography-electrospray ionization-mass spectrometry (LC-ESI-MS) using a 6520 Accurate Q-TOF LC/MS instrument (Agilent, Santa Clara, CA, USA).

### 4.3. Radiolabeling

Site-specific radiolabeling of the three new variants with [^99m^Tc][Tc=O]^+^ using the C-terminal amino-acid based cysteine-containing chelators was performed similarly to methodology described earlier by Oroujeni et al. [29]. A freeze-dried labeling kit containing 75 mg of tin (II) chloride dihydrate (Fluka Chemika, Buchs, Switzerland), 5 mg of gluconic acid sodium salt (Celsus Laboratories, Geel, Belgium), and 100 µg of EDTA (Sigma-Aldrich, Darmstadt, Germany) was prepared for the labeling.

In a general labeling procedure, one freeze-dried kit was reconstituted in 100 µL PBS, mixed with 75 µg of the DARPin G3 in PBS (100 µL) and 100 µL of generator eluate containing ^99m^Tc-pertechnetate (150–300 MBq) was added. The mixture was incubated at 60 °C for 30 min (variants G3-G_3_C and G3-(G_3_S)_3_C) or 60 min (variant G3-E_3_C). After incubation, radiolabeled DARPins were isolated using size-exclusion chromatography on disposable NAP-5 columns, pre-equilibrated and eluted with PBS containing 20 µg/mL of tin (II) chloride dihydrate. Radio-iTLC analysis of [^99m^Tc]Tc-labeled proteins was performed in PBS. In this system, the ^99m^Tc-radiolabeled DARPin-G3 and the reduced-hydrolyzed technetium (RHT) remained at the application point, and all forms of free radionuclides (including [^99m^Tc]TcO_4_^−^ [^99m^Tc]Tc-gluconic acid) migrated with the solvent front.

The initial experiments were performed without pre-reduction of the DARPins. Due to the low yield in this case, further labeling experiments included pre-reduction of spontaneously formed intermolecular disulfide bonds for all new variants. A solution of DTT (150-fold molar excess, 15 mg/mL in degassed 0.01 M PBS, pH 7.4) was mixed with a DARPin (500 µg, 2 mg/mL in 0.02 M PBS, pH 7.4) to obtain a final DTT concentration of 18 mM. The mixture was incubated at 40 °C for 60 min. Purification of reduced DARPin-G3 was performed using a NAP-5 column equilibrated and eluted with PBS. The concentration of the purified protein in fractions was measured using a NanoDrop OneC (Thermo Scientific, Waltham, MA, USA). Thereafter, the reduced DARPin-G3 was divided into aliquots, 75 µg in 100 µL PBS each, and stored at −20 °C before labeling.

To evaluate stability, solutions of the ^99m^Tc-radiolabeled DARPin-G3 (20 µL, 1.5 µg) were incubated in PBS (80 µL) for 1, 2, and 4 h at 37 °C. The test was performed in duplicate.

Site-specific radiolabeling of (HE)_3_-G3 using [^99m^Tc][Tc(CO)_3_]^+^ (tricarbonyl technetium) via the N-terminal amino acid sequence (HE)_3_ was adapted and performed as described earlier by Vorobyeva et al. [25]. Briefly, the [^99m^Tc]TcO_4_^-^, eluate in 1000 μL of 0.9% NaCl (2–4 GBq) was added to a CRS kit vial, followed by incubation at 100 °C for 30 min to generate the [^99m^Tc]Tc(CO)_3_]^+^ precursor and was allowed to cool at room temperature for 10 min. Then, 100 μL [^99m^Tc]Tc(CO)_3_^+^ from the CRS reaction mixture (100–200 MBq) was added to 40 μg (2.75 nmol) of (HE)_3_-G3 in 9 μL of PBS followed by addition of 100 μL 0.1 M HCl to pH 7.5–8. The reaction mixture was incubated at 60 °C for 60 min. After incubation, a 1000-fold molar excess of histidine (2.75 μmol, 425 μg, 21.5 μL of 20 mg/mL in PBS) was added to the mixture and further incubated for 15 min at 60 °C. Radiolabeled (HE)_3_-G3 was purified using NAP-5 columns (Cytiva, Amersham, UK) pre-equilibrated and eluted with PBS. Radiochemical yield and purity were measured using radio-iTLC in PBS. The radiolabeled (HE)_3_-G3 and RHT colloid remained at the application point, while [^99m^Tc]TcO_4_^−^, [[^99m^Tc]Tc(CO)_3_], and its complex with histidine migrated with the solvent front.

### 4.4. In Vitro Studies

Based on previous literature, we tested the binding specificity of all variants of ^99m^Tc-labeled DARPin-G3 molecules using cell lines with three different levels of HER2 expression (the ovarian cancer cell line SKOV-3 and the breast cancer cell line SK-BR-3 with high expression levels, and the prostate cancer cell line PC-3 with low expression) [42]. Cells were seeded in 6-well plates at a density of 6 × 10^5^ cells per dish one day before the experiment. Two sets of dishes were used for each cell line. A 100-fold excess of unlabeled DARPin-G3 protein (500 µL) was added to the control group of cells (3 dishes) to saturate HER2 receptors 30 min before the addition of the labeled compound. To the other three dishes, an equal volume of complete media was added. Thereafter, a solution of ^99m^Tc-labeled DARPins was added to each dish to reach a concentration of 1 nM. The dishes were incubated for 1 h at 37 °C. After incubation, the medium was collected, the cells were washed with PBS, and these solutions were pooled. The cells were then detached by trypsinization and collected. The dishes were washed with PBS and the solutions were added to the cell suspensions. The activity in the fractions containing medium or cells was measured using a gamma-spectrometer and the percentage of cell-associated activity was calculated. The experiments were performed in triplicates.

Cellular processing and internalization of all variants of ^99m^Tc-labeled DARPin G3 were evaluated using SKOV-3 cells. The cells (3 dishes per time point, 10^6^ cells per dish) were continuously incubated with 1 nM ^99m^Tc-labeled proteins at 37 °C. During the incubation, the internalized fraction was determined periodically by an acid wash method previously described for affibody molecules by Wållberg and Orlova [42]. The membrane-associated DARPins were removed from the cells by treatment with a 4 M urea solution in a 0.2 M glycine buffer, pH 2.0, for 5 min on ice. The solution was collected and its activity was measured. The cell debris containing the internalized conjugates was detached by treatment with 1 M NaOH for 30 min at 37 °C. The radioactivity in the fractions was measured, and the percentage of total cell-associated, membrane-associated, and internalized radioactivity was calculated. To validate this protocol for new variants of the ^99m^Tc-labeled DARPin G3, the cells were incubated on ice (when no internalization should take place) for 2 h with labeled DARPins molecules and then the internalization measurement was performed as described above.

Equilibrium dissociation constants (K_D_) were determined using a saturation assay. A set of four cell culture dishes with SKOV-3 cells was prepared for each concentration of the tested protein. Three dishes were used to determine a specific binding and one cell culture dish was used for the determination of nonspecific binding by receptor blocking. The cells were seeded one day before the experiment. Immediately before measurements, the medium was discarded and the cells were washed with PBS. Eight labeled protein concentrations (ranging from 0.22 to 40 nM) were prepared. Each control dish was pre-saturated with 500 µL of non-labeled protein at a concentration of 2000 nM. Only media with serum (the same volume) was added to other dishes of the set, and the cells were incubated in a humidified incubator (5% CO_2_, 37 °C) for 30 min. The required concentration of a labeled DARPin was added to each set of cells (4 °C, 500 µL per dish) and the cells were incubated at 4 °C for 4 h. The medium was discarded and the cells were washed with PBS (×5) before a trypsin–EDTA solution (500 µL per dish) was added, and cells were additionally incubated for 10 min. Detached cells were diluted with 500 µL of complete medium, re-suspended and transferred to fraction tubes. A 50 µL sample was taken from each tube for cell counting. The radioactivity of the cells and the labeled protein standards were measured using an automated gamma counter. The real added radiolabeled protein concentrations were calculated for each data point using the highest concentration. Then cell-associated radioactivity as pmol/10^5^ cells for every sample was calculated. B_max_ (maximum number of binding sites per cell) and K_D_ were calculated using Prism 9 for Windows (GraphPad Sofware, San Diego, CA, USA).

### 4.5. Animal Studies

To evaluate the biodistribution of the ^99m^Tc-labeled DARPin-G3 variants, 16 female CD1 mice were divided into four groups of four animals each. The average animal weight was 29.2 ± 2.3 g. The mice were intravenously (i.v.) injected with 5 µg (60 kBq) of [^99m^Tc]Tc-G3-G_3_C, [^99m^Tc]Tc-G3-(G_3_S)_3_C, [^99m^Tc]Tc-G3-E_3_C, or [^99m^Tc]Tc-(HE)_3_-G3 in 100 µL PBS with 1% BSA containing 10 µg/mL of tin (II) chloride dihydrate. Anaesthetized mice were sacrificed by cervical dislocation. The blood, other organs, and tissues of interest were collected and weighed, and the activity was measured using an automated gamma-counter. The activity uptake was calculated as the percentage of injected dose per gram of sample (%ID/g).

To evaluate the targeting properties, the biodistribution of ^99m^Tc-labeled DARPins (variants G3-G_3_C, G3-(G_3_S)_3_C and (HE)_3_-G3) was directly compared in a single batch of Nu/j mice bearing SKOV-3 xenografts with high HER2 expression. To evaluate if the tumor uptake was dependent on HER2 expression levels, the uptake of these DARPins was also measured in PC-3 xenografts with low HER2 expression. Female Nu/j mice were subcutaneously implanted with 10^7^ SKOV-3 or the same amount of PC-3 cells in 100 μL media. The experiments were performed four weeks after SKOV-3 cell implantation and two weeks after PC-3 cell implantation. The average animal weight at the time of the experiment was 24.8 ± 1.9 g. The average tumor weight was 0.5 ± 0.2 and 0.05 ± 0.02 g for SKOV-3 and PC-3 xenografts, respectively. A group of four mice was used for each construct. The mice were injected with 10 µg of ^99m^Tc-labeled DARPin-G3 (60 kBq, 100 µL in PBS with 1% BSA containing 10 µg/mL of tin (II) chloride dihydrate) into the tail vein. The biodistribution of ^99m^Tc-labeled DARPin-G3 was measured 4 h after injection. This time point was selected because a clinical study [26] has demonstrated that imaging 4 h after injection provides the best discrimination between HER2-positive and HER2-negative malignant tumors. The measurement of biodistribution in tumor-bearing mice was performed in the same way as in CD1 mice (see above).

Imaging of mice bearing SKOV-3 and PC-3 xenografts was performed using a Siemens E.Cam 180 scanner equipped with a high-resolution low-energy collimator (Siemens, Germany). For imaging of HER2 expression, mice were injected with [^99m^Tc]Tc-(HE)_3_-G3 (10 μg, 8.2 MBq), [^99m^Tc]Tc-G3-G_3_C (10 μg, 8.2 MBq), or [^99m^Tc]Tc-G3-(G_3_S)_3_C (10 μg, 8.4 MBq). At 4 h pi, anesthetized mice were sacrificed by cervical dislocation and placed on the collimator of the gamma camera. This form of euthanasia was necessary because a clinical camera was used for imaging, and using gas anesthesia during imaging was problematic. Moreover, gas anesthesia mediated euthanasia often causes mice to urinate. This results in the elimination of high urinary bladder activity, and a frequent artifact in preclinical imaging studies is avoided. Images were acquired for 30 min and stored in a 1024 × 256 pixel matrix. Animal contours were derived from digital photographs and were superimposed over the gamma-camera image to simplify interpretation.

## 5. Conclusions

The use of cysteine-containing peptide-based chelators enables stable labeling of the DARPin G3 with ^99m^Tc. The labeled conjugates demonstrated specific and high affinity binding to HER2-expressing human cancer cells in vitro and specific accumulation in HER2-expressing xenografts in vivo. However, [^99m^Tc]Tc-(HE)_3_-G3 provides better contrast between tumors and most frequent metastatic sites of major HER2-expressing cancers and is therefore more suitable for clinical applications.

## Figures and Tables

**Table 1:** Results of the LC-MS analysis of non-labeled DARPin G3 variants having the amino acid-containing chelating sequences G_3_C, E_3_C, and (G_3_S)_3_C at the C-terminus.

Construct	Mw_calc_ (Da)	Mw_obs_ (Da)
G3-G_3_C	13,529.3	27,057.3 (dimer) 13,529.7 (monomer)
G3-(G_3_S)_3_C	14,075.6	28,149.8 (dimer) 14,075.9 (monomer)
G3-E_3_C	13,745.5	27,489.4 (dimer) 13,745.7 (monomer)

**Table 2:** Radiochemical yield, isolated yield, radiochemical purity, and maximum specific activity of ^99m^Tc-labeled G3 variants. Experiments were performed in duplicates. * Radiochemical yield is based on iTLC analysis of the labeled constructs before purification. Isolated yield is defined as the percentage of activity in the reaction vial before purification, which was recovered in the high molecular weight fraction after purification.

Variant	Radiochemical Yield, % *	Isolated Yield, % *	Radiochemical Purity, %	Maximum Specific Activity, MBq/μg
[^99m^Tc]Tc-G3-G_3_C	98 ± 1	85 ± 2	100 ± 0	5.1
[^99m^Tc]Tc-G3-(G_3_S)_3_C	98 ± 1	87 ± 2	100 ± 0	4.8
[^99m^Tc]Tc-G3-E_3_C	98 ± 1	82 ± 2	100 ± 0	4.5
[^99m^Tc]Tc-(HE)_3_-G3	75 ± 2	62 ± 3	98 ± 1	4.8

**Table 3:** Summary of findings from a direct (saturation) receptor-binding curve for the binding of [^99m^Tc]Tc-labeled G3 to SKOV-3 cells. The K_D_ and B_max_ values derived from the specific binding curves.

	[^99m^Tc]Tc-G3-G_3_C	[^99m^Tc]Tc-G3-(G_3_S)_3_C	[^99m^Tc]Tc-G3-E_3_C	[^99m^Tc]Tc-(HE)_3_-G3
K_D_, nmol	3.3 ± 0.5	4.1 ± 0.6	5.0 ± 0.9	1.9 ± 0.2
B_max_, receptors/cell	1.59 ± 0.07 × 10^6^	1.49 ± 0.01 × 10^6^	1.03 ± 0.06 × 10^6^	1.22 ± 0.03 × 10^6^

## Data Availability

The data generated during the current study are available from the corresponding author upon reasonable request.
